# The Association between Social Integration and Utilization of Essential Public Health Services among Internal Migrants in China: A Multilevel Logistic Analysis

**DOI:** 10.3390/ijerph17186524

**Published:** 2020-09-08

**Authors:** Jing Liang, Yujia Shi, Mohammedhamid Osman, Bhawana Shrestha, Peigang Wang

**Affiliations:** 1 School of Health Sciences, Wuhan University, 115 Donghu Road, Wuhan 430071, China; liang0827@whu.edu.cn (J.L.); jebibet@gmail.com (M.O.); bhavanashrestha75@gmail.com (B.S.); 2 School of Philosophy, Zhongnan University of Economics and Law, South Nanhu Road, Wuhan 430073, China; 201912160043@stu.zuel.edu.cn

**Keywords:** internal migrants, social integration, health records, health education, multilevel analysis

## Abstract

This study investigated the association between social integration and utilization of essential public health services among internal migrants. Data were from the 2017 China Migrants Dynamic Survey. Social integration was measured through four dimensions: economic integration, structural integration, sociocultural adaptation, and self-identity. Multilevel logistic regressions were used taking into account heterogeneity in the level of regional development. The utilization of health records and health education was less than 40% and varied widely across regions. Social integration was related to a higher likelihood of utilization of health records and health education. Moreover, sociocultural adaptation had a stronger effect on the utilization of health records in developed regions than in developing regions, and structural integration was strongly and positively related to the utilization of health education in developed regions. Hence, it appears that the relationship of some dimensions of social integration and utilization of essential public health services is moderated by the level of economic development. Promoting structural integration and sociocultural adaptation could strongly improve utilization of essential public health services in developed regions.

## 1. Introduction

As a vulnerable and specific group, migrants often experience a lack of access to health care services [1]. The UN 2030 Agenda for the Sustainable Development calls for empowering vulnerable groups such as internal migrants to reduce inequalities and to ensure healthy lives for all ages [2]. In recent years, the health status of migrants has received attention in global public health circles [3]. The World Health Organization has been promoting the health of migrants to achieve universal health coverage [4]. According to reports, China has the world’s largest (30%) internal immigration [5], estimated at 245 million in 2017. The Chinese government attaches great importance to the health of internal migrants, and some measures have been taken to promote the health of internal migrants.

A national program to provide free access to essential public health services (EPHS) to all Chinese people was launched in 2009 [6]. At present, these services comprise 14 components including key items such as establishing health records and receiving health education. These two items could be used to understand and track the health status and health literacy of individuals [7]. However, the utilization of health records and health education among internal migrants is still lower than that of local residents. In 2015, for instance, less than 30% of migrants had established health records, far below the local residents [8], and more than half of them did not receive any health education on prevention of infectious and noncommunicable diseases [9]. Promotion of health among internal migrants is obstructed by low utilization of health services [10].

Several studies indicated that internal migrants who are male with low income and education were less likely to access healthcare services. Similarly, migration to the eastern region, interprovincial migration, and less migration time were related to inadequate use of healthcare services [11,12,13]. However, these studies did not take into account one fundamental problem of migrant-social integration. Social integration is the process of incorporating newcomers into the local social structure. Internal migrants ought to align with the local values and norms. Successful assimilation helps migrants build up social capital and improve individual well-being [14].

Many studies have found that social integration has powerful effects on health and health care services [15,16,17]. As a multidimensional variable, different proxy measures have been used to quantify social integration [14,18,19]. Jesus [17], for instance, used community involvement as a proxy for social integration and found that social integration reduced the association between stigma and sexual risk behavior. Berkman [20] employed a social integration index calculated from three dimensions (marital status, contacts with close friends and family, and affiliation with voluntary associations) and found that social integration was an important predictor of mortality. Lin [21] reported two aspects of social integration, economic integration, and self-identity, which were significantly associated with health status. Furthermore, Kathleen [22] indicated that social integration strengthened HIV prevention efforts, where social integration was measured using the duration of residence in the community, ability to speak the local language, interaction with the local community, and participation in social events. Although there is no unified definition of social integration, economic integration together with acculturation, structural integration, and identity integration, are the main dimensions to understand and explain social integration of migrants [23,24].

To our knowledge, one study has found the association between social integration and the establishment of health records among elderly migrants in China [25]. However, it is uncertain whether this association still exists in young migrants. Different provinces have different levels of economic development in China. In regions with good economic development, high-quality health resources, higher management levels, and more rational resource planning are expected [26], but fewer utilization of public health services among internal migrants has been discovered [27]. It is not clear whether there is a positive association between social integration and the utilization of essential public health services across different regions, which may suggest a research gap in the current literature.

Taking into account the hierarchical structure of data, our study uses the multilevel model to explore the association between social integration and the utilization of essential public health services among internal migrants in China, particularly health records and health education on the protection of infectious and noncommunicable chronic diseases. We also seek to test whether the effect of the regional development moderated the relationship.

## 2. Materials and Methods

### 2.1. Data and Study Design

The China Migrants Dynamic Survey (CMDS) was conducted by the National Health and Family Planning Commission of China to monitor the flow of internal migrants, housing, employment, and the utilization of health services. The CMDS is a nationally representative cross-sectional survey of internal migrants (15–59 years old), which has been conducted annually since 2009. Internal migrants are people who migrate from their hometown to other counties/districts/cities for at least one month but without their household registration (Hukou) (excluding students, soldiers, and households where one of the spouses has the local hukou). The sample was drawn using a stratified multi-stage random sampling method with the Probability Proportional to Size (PPS) approach. To be specific, 31 provincial units were selected first. Towns/neighborhoods were then selected by using PPS method. Subsequently, villages/communities were also selected by using the same method. A list containing 100 migrants in each village/community was provided by local officials, and 20 migrants in each village/community were randomly selected and investigated by trained investigators. We used data from the 2017 CMDS, which surveyed 169,989 individuals covering 348 cities and 10,300 communities. Internal migrants aged 18 and older who answered all survey questions were retained. The final sample in the study was 154,008. All the participants gave their informed written consent for participation prior to the face-to-face interview. The raw data of CMDS 2017 used in this study was approved by the National Population and Family Planning Commission of China. Data and details are available on the website http://www.chinaldrk.org.cn/wjw/#/application/index. The analysis of public access data was exempted by the Ethics Committee of the School of Health Sciences, Wuhan University, China.

### 2.2. Dependent Variables

Three indicators were used to measure the utilization of essential public health services by internal migrants: health records, health education on the prevention of infectious diseases (ID), and health education on the prevention of noncommunicable chronic diseases (NCD). The utilization of health records was assessed using the question “Have you established a local health record?” with response options “Yes”; “No, but heard of it”; “No, never heard of it”; and “I don’t know”. It was recoded into a dichotomous variable (1 = “Yes” vs. 0 = “No”) at last. The questions “Have you received health education on prevention of infectious diseases/noncommunicable disease in your local residence?” with “Yes’’ and ‘’No” response options were used to measure the utilization of health education. The answers included “Yes’’ and ‘’No”.

### 2.3. Independent Variables

#### 2.3.1. Social Integration

Social integration was measured by the index system presented by Yang [18] and Zhou [19], which includes economic integration, structural integration, sociocultural adaptation, and self-identity dimensions. Economic integration comprised two indicators: employment (employed vs. unemployed) and residential pattern (owning local house vs. not). Structural integration was measured by three indicators: social organizational participation, civil activities engagement, and local medical insurance (“Yes” = 1 vs. “No” = 0). On social organizational participation, participants were asked whether they participated in any labor union, volunteer association, alumni association, chamber of commerce of hometown, association of migrants from the same hometown, and other organizations; response options included “Yes” = 1 vs. “No” = 0. Civil activity engagement was also dichotomized into (1 = “Yes” vs. 0 = “No”) responses based on whether a participant had been involved in management of social public welfare and other community activities. Sociocultural adaptation was measured by local friends, through the question “Who do you hang out with most after work hours in the local residence?” Participants answering “local friends” was defined by ‘’Yes’’; otherwise, it was “No”. The question “Do you plan to settle in a local residence?” (1 = “Yes” vs. 0 = “No”) was used as a measure of self-identity.

#### 2.3.2. Provincial-Level Variable

The regional development was indexed by the Human Development Index (HDI), which captures an average achievement in key dimensions of human development. HDI is a composite index based on three dimensions: life expectancy at birth, literacy rate, and GDP per capita [28]. The HDI scores range from 0 to 1, where HDI > 0.8 is defined as developed regions and the rest are defined as developing regions (1 = “developed regions” vs. 0 = “developing regions”).

### 2.4. Control Variables

A wide variety of individual and migration characteristics were controlled in our study: Age (two groups: <35 years old and ≥35 years old), gender (two groups: male and female), marital status (two groups: currently married and single), education (three groups: primary school or below, junior high school, and senior high school or above), and family monthly income divided into four quartiles (Q1to Q4), with Q1 and Q4 indicating the lowest and the highest incomes, respectively. Household registration (hukou) (two groups: Agriculture and non-agriculture) were included in the variables on individual characteristics. The length of stay in years (a continuous variable) and the range of migration (three groups: Across provinces, across cities within a province, and across counties within a city) were included in the variables on migration characteristics.

### 2.5. Data Analysis

Descriptive statistics were used to summarize characteristic variables related to individual, migration, provincial-level, social integration, health records, and health education. We performed chi-square test on social integration and dependent variables. We also used multilevel logistic regressions to assess the effect of social integration on the utilization of health records and health education among internal migrants. First, we ran an empty model of multilevel logistic regressions, and the intra-class correlation coefficient for health records and health education on the prevention of ID and NCD were 17.75%, 15.31%, and 13.13%, respectively (Supplementary Materials, Table S1), which indicated that there was a certain degree of intergroup heterogeneity in the analyzed data. Hence, multilevel logistic analysis was necessary [29]. Second, we included social integration variables and control variables in model 1. Finally, we included the second-level variable (the regional development) and the interaction terms between the second-level variable and social integration variables in model 2 based on model 1. The difference between the two models can be illustrated as follows: The first-level equation was the same for the two models:

Level 1:

(1) $$log\left(\frac{P_{ij}}{1-P_{ij}}\right)=\beta_{0j}+\beta_{1j}x_{ij}$$

$P_{ij}$ represents the probability of outcome variable (i.e., health record and health education on prevention of ID and NCD) for respondent i  in province j.$\;\beta_{0j}$ is the intercept for province j.$\;\beta_{1j}$ is the coefficient for social integration in province j.$\;x_{ij}$ represents social integration (i.e., economic integration, structural integration, sociocultural adaptation, and self-identity).

Model 1 used the following formulas for the two coefficients ($\beta_{0j}$ and $\;\beta_{1j}$) in Equation (1):

Level 2:

$$\beta_{0j}=r_{00}+u_{0j}\;\;\beta_{1j}=r_{10}+u_{1j}$$

Model 2 additionally analyzed the effect of the regional development. The two coefficients ($\beta_{0j}$ and $\;\beta_{1j}$) in the Equation (1) were specified as follows:

Level 2:

$$\beta_{0j}=r_{00}+r_{01}W_{1j}\;+u_{0j}\;\beta_{1j}=r_{10}+r_{11}W_{1j}\;+u_{1j}$$

$r_{00}$ represents the mean logit of the outcome variable in the intercept, $u_{0j}$ represents the mean logit random variation at the group level in the intercept.$\;r_{10}$ represents the mean effect in predicting the outcome variable in slope, $u_{1j}$ represents the deviation from it at the group level in the slope. $W_{1j}$ represents the regional development j.$\;r_{01}$ and $r_{11}$ represent the effect of the societal development in predicting the outcome variable in the intercept and slope, respectively. All models were adjusted control variables, which had been centralized by the grand mean. All statistical analyses were conducted using SAS 9.4 (SAS Institute Inc., Cary, NC, USA). The significant level was set at *p* < 0.05.

## 3. Results

### 3.1. Individual, Migration, and Provincial Characteristics of Internal Migrants

Table 1 presents the characteristics distributions of internal migrants. Among the 154,008 migrants, 51.51% were male. The average age was 37.12 years (SD) = 10.96; 60.46% of internal migrants received education level of junior or below; 83.86% were married; and 77.37% had agriculture hukou. The average monthly income of a family was 3771.90 RMB (SD = 3041.62); 48.44% were across provinces, and the average time in inflow area was 6.98 years (SD = 6.04); 16.23% of them stayed in developed regions (Beijing, Tianjin, Shanghai, Jiangsu, Zhejiang, and Liaoning). The education and economic levels of internal migrants in developed regions were higher than those in developing regions.

### 3.2. Social Integration and the Utilization of EPHS among Internal Migrants

Overall, 30.03% of internal migrants established health records, and 33.67% and 37.36% received health education on the prevention of ID and NCD, respectively. The utilization rates of these services in developing regions were higher than that in developed regions. On economic integration, 82.07% were employed and 30.83% had a local house. Regarding structural integration, 44.93% participated in social organizations, 42.52% were engaged in civil activities, and 22.71% had local medical insurance. For sociocultural adaptation, 37.82% of internal migrants had the most contact with local friends after work. As for self-identity, 83.43% of them decided to settle in their current residence in the future. Generally, migrants to developed regions recorded higher percentages in most indicators of social integration (Table 2).

There was a higher proportion of respondents having a local house, participating in organizations, engaging in civil activities, having local medical insurance, having local friends, and willing to settle among those who had established health records and received health education information on prevention of ID and NCD (*p* < 0.001) (Table 3).

### 3.3. Associations between Social Integration and the Utilization of EPHS among Internal Migrants

Table 4 displays the multilevel logistic regression analysis of the association between social integration and the utilization of health records. Taking into account the hierarchical structure of data and adjusting for control variables, social integration was associated with the utilization of health records. Internal migrants who were employed (OR = 0.955, 95% CI: 0.921–0.991) were less likely to establish health records, but those who had a local house (OR = 1.082, 95%CI: 1.047–1.118), participated in social organizations (OR = 1.618, 95%CI: 1.576–1.660), were engaged in civil activities (OR = 1.355, 95%CI: 1.320–1.390), had local medical insurance (OR = 1.321, 95%CI: 1.283–1.361), had local friends (OR = 1.118,95%CI: 1.085–1.151), and who were willing to settle (OR = 1.247, 95% CI: 1.204–1.291) were more likely to establish health records (Table 4, model 1). We further examined whether the cross-regional variation was moderated by the regional development (Table 4, model 2) and found that sociocultural adaptation was (OR = 1.201, 95% CI: 1.035–1.394) more strongly predictive of the utilization of health records in developed regions than in developing regions.

Table 5 presents the same set of analyses to explore the relationship between social integration and the utilization of health education. These dimensions of economic integration structural integration, sociocultural adaptation, and self-identity affected positively the utilization of health education on the prevention of ID and NCD (Table 5, model 1a and model 1b). We also further examined whether the association was moderated by regional development and found that structure integration was moderated by regional development. Specifically, internal migrants who had participated in social organizations (OR = 1.145, 95%CI: 1.061–1.235) and were engaged in civil activities (OR = 1.120, 95%CI: 1.020–1.229) were more strongly and positively related to the utilization of health education on prevention of ID in developed regions than in developing regions (Table 5, model 2a). Those who were engaged in civil activities (OR = 1.104, 95%CI: 1.014–1.203) were more strongly predictive of health education on prevention of NCD in developed regions than those living in developing regions (Table 5, model 2b).

## 4. Discussion

The aim of this study was to examine the relationship between social integration and the utilization of EPHS among internal migrants and to assess the heterogeneous effects of social integration across regional development in China, using multilevel logistic models.

Consistent with the previous study [27], the utilization of health records and health education among internal migrants was less than 40%, whereas 90% of local residents had access to these services [30]. The utilization rate in developed regions was lower than that in developing regions, which is inconsistent with the local economic level and health resource allocation [31]. This shows that the utilization of EPHS requires active participation of residents. There are significant differences in lifestyle, health beliefs, and risk factors between internal migrants and local residents [32], which could have an impact on migrants’ access to health systems and participation in prevention services.

By using a more comprehensive dimension to measure social integration, our study found that it was related to a higher likelihood of utilizing EPHS among internal migrants. For economic integration, owning a local house is a sign that their incomes are similar to those of local residents. As new citizens, they might participate in these local social activities and have better access to EPHS [33]. For structural integration, engagement in social organizations or social activities encourages information exchange and opinion sharing with others within the communities and enhances the sense of belonging [9,34,35]. Having local health insurance could help migrants understand local health resources [36], including hospitals and community health services, which could improve people’s equal access to health services [37]. For sociocultural adaptation, local friends could provide social support to internal migrants and could help them be informed of local and useful health information, which is important for elderly and youth migrants to use health care services [10,25,38,39]. For self-identity, migrants with long-term willingness to settle were of higher possibility to participate in social activities [40]. Internal migrants have higher employment rates, less time to participate in social organizations or activities, and weaker native networks, which could make access to health information resources difficult [41,42]. These results have important policy relevance for local government to promote more social integration among internal migrants and to encourage health education in the workplace and participation in community activities.

It is worth noting that the association between social integration and the utilization of EPHS varied across regions, with a greater effect in the developed regions. To the best of our knowledge, these are novel findings because, in the literature on social integration and utilization of health services, the role of regional development has often been controlled in analyses but is rarely examined as a factor that moderates the association between social integration and utilization of health services. Prior research has suggested that mental health implications of social integration might differ across societies [43], and our research extended this pattern to the important outcome variables in the utilization of healthcare services.

In addition, the utilization of health education on the prevention of ID was lower than that of NCD in both developed and developing regions. Although communicable diseases declined significantly, the problems did not disappear [44]. If infectious diseases occur, the management of internal migrants could be difficult and may bring serious risks to the public. Migration is often accompanied by unhealthy exposures and environments, increasing the risk for HIV infection and tuberculosis [45,46]. In the current coronavirus disease 2019 pandemic, migrants are at higher risk if they face exclusion and multiple barriers to health care [47]. Therefore, the local government needs to strengthen health follow-up management and health education on epidemic prevention and control for internal migrants.

There were several potential limitations to this study. First, as there is no unified definition of social integration, we used four dimensions to measure social integration based on the literature but there are still biases of reliability and validity caused by incomplete dimensions. Second, the responses given by self-reported measures could be affected by response bias, and we will work with community health services to reduce response bias for future research. Third, the survey did not collect information on the health of internal migrants, which could also be associated with the utilization of health care services [48]. We can consider internal migrants aged 15 to 59 as a relatively healthy population if we consider the “salmon bias” (healthier individuals are more likely to migrate) [49]. Fourth, although we only focus on internal migration, social integration is equally important for international migration. Last, the cross-sectional design of the study cannot predict the causal relationship between variables; further research is needed to confirm the causal relationship between social integration and the utilization of health service.

## 5. Conclusions

In summary, social integration may be associated with the utilization of EPHS among internal migrants in China, and this association is moderated by regional development. The findings imply that promotion of social integration might be more effective on the utilization of health records and health education in developed regions than in developing regions, mainly through structural integration and sociocultural adaptation. To achieve the equalization of essential public health services, more interventions should be implemented to enhance social integration among internal migrants, especially in developed regions. Participation in social organizations and community activities should be encouraged among internal migration.

## Figures and Tables

**Table 1:** Characteristics of internal migration in 2017 (N = 154,008).

Variables	All Sample	The Developed Regions	The Developing Regions
	*N* (%)	*N* (%)	*N* (%)
Age(years)			
>35	72,924 (47.35)	14,110 (47.36)	58,814 (47.35)
≤35	81,084 (52.65)	15,683 (52.64)	65,401 (52.65)
Gender			
Male	79,337 (51.51)	15,009 (50.38)	64,328 (51.79)
Female	74,671 (48.49)	14,784 (49.62)	59,887 (48.21)
Education attainment			
Primary school and below	26,315 (17.09)	4021 (13.50)	22,294 (17.95)
Junior high school	66,793 (43.37)	12,035 (40.40)	54,758 (44.08)
Senior high school and above	60,900 (39.54)	13,737 (46.11)	47,163 (37.97)
Marital status			
Married	129,154 (83.86)	25,420 (85.32)	103,734 (80.32)
Single	24,854 (16.14)	4373 (14.68)	20,481 (16.49)
Family monthly income			
Q1 (lowest)	45,554 (29.58)	7278 (24.43)	38,276 (30.81)
Q2	41,344 (26.85)	7023 (23.57)	34,321 (27.63)
Q3	28,968 (18.81)	5319 (17.85)	23,649 (19.04)
Q4 (highest)	38,142 (24.77)	10,173 (34.15)	27,969 (22.52)
Hukou			
Agriculture	119,160 (77.37)	21,533 (72.28)	97,627 (78.60)
Non-agriculture	34,848 (22.63)	8260 (27.72)	26,588 (21.40)
The length of stay in years *	6.98 ± 6.04	7.88 ± 6.34	6.76 ± 5.94
The range of migration			
Across counties within a city	27,914 (18.13)	858 (2.88)	27,056 (21.78)
Across cities within a province	51,489 (33.43)	3682 (12.36)	47,807 (38.49)
Across provinces	74,605 (48.44)	25,253 (84.76)	49,352 (39.73)
The regional development			
The developed regions	29,793 (19.35)	-	-
The developing regions	124,215 (80.65)	-	-

Note: *: Mean, SD. ID: infectious diseases; NCD: noncommunicable chronic disease.

**Table 2:** Social integration and the utilization of essential public health services (EPHS) among internal migrants.

Variables	All Sample	The Developed Regions	The Developing Regions
	*N* (%)	*N* (%)	*N* (%)
Health records			
Yes	46,244 (30.03)	4695 (15.76)	41,549 (33.45)
No	107,764 (69.97)	25,098 (84.24)	82,666 (66.55)
Health education on the prevention of ID			
Yes	51,857 (33.67)	5268 (17.68)	46,589 (37.51)
No	102,151 (66.33)	24,525 (82.32)	77,625 (62.49)
Health education on prevention of NCD			
Yes	57,692 (37.36)	6766 (22.71)	50,926 (41.00)
No	96,316 (62.54)	23,027 (77.29)	73,289 (59.00)
Social integration			
Economic integration			
Employment			
Yes	126,401 (82.07)	24,854 (83.42)	101,547 (81.75)
No	27,607 (17.93)	4939 (16.58)	22,668 (18.25)
Having a local house			
Yes	47,478 (30.83)	9111 (30.58)	38,367 (30.89)
No	106,530 (69.17)	20,682 (69.42)	85,848 (69.11)
Structural integration			
Organizational participation			
Yes	69,199 (44.93)	11,824 (39.69)	57,375 (46.19)
No	84,809 (55.07)	17,969 (60.31)	66,840 (53.81)
Civil engagement			
Yes	65,486 (42.52)	12,150 (40.78)	53,336 (42.94)
No	88,522 (57.48)	17,643 (59.22)	70,879 (57.06)
Local medical insurance			
Yes	34,969 (22.71)	9207 (30.90)	25,762 (20.74)
No	119,039 (77.29)	20,586 (69.10)	98,453 (79.26)
Sociocultural adaptation			
Having local friends			
Yes	58,245 (37.82)	9107 (30.57)	49,138 (39.56)
No	95,763 (62.18)	20,686 (69.43)	75,077 (60.44)
Self-identity			
Settlement willingness			
Yes	128,485 (83.43)	26,360 (88.48)	102,125 (82.22)
No	25,523 (16.57)	3433 (11.52)	22,090 (17.78)

Note: ID: infectious diseases; NCD: noncommunicable chronic disease.

**Table 3:** Cross-tabulation between social integration and utilization of EPHS.

Variables	Health Records			Health Education on the Prevention of ID			Health Education on the Prevention of NCD		
	Yes (*N*/%)	No (*N*/%)	χ2/*p*	Yes (*N*/%)	No (*N*/%)	χ2/*p*	Yes (*N*/%)	No (*N*/%)	χ2/*p*
Economic integration									
Employment			χ^2^ = 85.35			χ^2^ = 153.98			χ^2^ = 26.03
Yes	37,317 (80.70)	89,084 (82.67)	*p* < 0.001	43,444 (83.78)	82,957 (81.21)	*p* < 0.001	47,722 (82.72)	78,679 (81.69)	*p* < 0.001
No	8927 (19.30)	18,680 (17.33)		8413 (16.22)	19,194 (18.79)		9970 (17.28)	17,637 (18.31)	
Having a local house			χ^2^ = 864.78			χ^2^ = 72.93			χ^2^ = 248.43
Yes	16,699 (36.11)	30,779 (28.56)	*p* < 0.001	16,718 (32.24)	30,760 (30.11)	*p* < 0.001	19,168 (33.22)	28,310 (29.39)	*p* < 0.001
No	29,545 (63.89)	76,985 (71.44)		35,139 (67.76)	71,391 (69.01)		38,524 (66.78)	68,006 (70.61)	
Structural integration									
Organizational participation			χ^2^ = 2481.78			χ^2^ = 3905.91			χ^2^ = 4397.70
Yes	25,236 (54.57)	43,963 (40.80)	*p* < 0.001	29,066 (56.05)	40,133 (39.29)	*p* < 0.001	32,188 (55.79)	37,011 (38.43)	*p* < 0.001
No	21,008 (45.43)	63,801 (59.20)		22,791 (43.95)	62,018 (60.71)		25,504 (44.21)	59,305 (61.57)	
Civil engagement			χ^2^ = 1902.09			χ^2^ = 3196.51			χ^2^ = 3805.19
Yes	23,542 (50.91)	41,944 (38.92)	*p* < 0.001	27,234 (52.52)	38,252 (37.45)	*p* < 0.001	30,324 (52.56)	35,162 (36.51)	*p* < 0.001
No	22,702 (49.09)	65,820 (61.08)		24,623 (47.48)	63,899 (62.55)		27,368 (47.44)	61,154 (63.49)	
Local medical insurance			χ^2^ = 794.29			χ^2^ = 239.49			χ^2^ = 310.18
Yes	12,624 (27.30)	22,345 (20.74)	*p* < 0.001	12,977 (25.02)	21,992 (21.53)	*p* < 0.001	14,501 (25.14)	20,468 (21.25)	*p* < 0.001
No	33,620 (72.70)	85,419 (79.26)		38,880 (74.98)	80,159 (78.47)		43,191 (74.86)	75,848 (78.75)	
Sociocultural adaptation									
Having local friends			χ^2^ = 996.55			χ^2^ = 813.36			χ^2^ = 884.31
Yes	20,243 (43.77)	38,002 (35.26)	*p* < 0.001	22,177 (42.77)	36,068 (35.31)	*p* < 0.001	24,558 (42.57)	33,687 (34.98	*p* < 0.001
No	26,001 (56.23)	69,762 (64.74)		29,680 (57.23)	66,083 (64.69)		33,134 (57.43)	62,629 (65.02)	
Self-identity									
Settlement willingness			χ^2^ = 391.70			χ^2^ = 86.40			χ^2^ = 155.24
Yes	39,904 (86.29)	88,581 (82.20)	*p* < 0.001	43,904 (84.66)	84,581 (82.80)	*p* < 0.001	49,011 (84.95)	79,474 (82.51)	*p* < 0.001
No	6,40 (13.71)	19,183 (17.80)		7953 (15.34)	17,570 (17.20)		8681 (15.05)	16,842 (17.49)	

Notes: ID: infectious diseases; NCD: noncommunicable chronic disease; EPHS: essential public health services.

**Table 4:** Multilevel logistic regression on the effects of social integration and establishing health records among internal migrants.

Variables	Model 1 AOR (95% CI)	Model 2 AOR (95% CI)
Economic integration		
Employment (ref. no)	0.955 (0.921–0.991) *	0.932 (0.860–1.011)
Having a local house (ref. no)	1.082 (1.047–1.118) ***	1.011 (0.917–1.114)
Structural integration		
Organizational participation (ref. no)	1.618 (1.576–1.660) ***	1.515 (1.415–1.621) ***
Civil engagement (ref. no)	1.355 (1.320–1.390) ***	1.353 (1.270–1.441) ***
Local medical insurance (ref. no)	1.321 (1.283–1.361) ***	1.335 (1.241–1.436) ***
Sociocultural adaptation		
Having local friends (ref. no)	1.118 (1.085–1.151) ***	1.140 (1.068–1.217) **
Self-identity		
Settlement willingness (ref. no)	1.247 (1.204–1.291) ***	1.248 (1.144–1.362) ***
The regional development		
Living in the developed regions (ref. the developing regions)		0.358 (0.203–0.632) **
Interaction between social integration and the regional development		
Employment × Developed regions		0.960 (0.801–1.150)
Having a local house × Developed regions		1.227 (0.979–1.539)
Organizational participation × Developed regions		1.094 (0.938–1.275)
Civil engagement × Developed regions		1.082 (0.938–1.247)
Local medical insurance × Developed regions		1.014 (0.866–1.187)
Having local friends × Developed regions		1.201 (1.035–1.394) *
Settlement willingness × Developed regions		0.940 (0.761–1.161)
−2LL	8,108,071.1	810,684.8

Notes: In model 1, we added social integration and control variables. In model 2, we added regional development and the interaction term between regional variable and social integration based on model 1. AOR: Adjusted odds ratio; CI: confidence interval; LL: log likelihood; significance level: *** *p* < 0.001, ** *p* < 0.01, and * *p* < 0.05.

**Table 5:** Multilevel logistic regression on the effects of social integration and receiving health education among internal migrants in China.

	Health Education on Prevention of ID		Health Education on Prevention of NCD	
	Model 1a	Model 2a	Model 1b	Model 2b
	AOR (95% CI)	AOR (95% CI)	AOR (95% CI)	AOR (95% CI)
Economic integration				
Employment (ref. no)	1.121 (1.081–1.163) ***	1.171 (1.091–1.257) ***	1.003 (0.969–1.039)	1.064 (0.991–1.142)
Having a local house (ref. no)	1.004 (0.972–1.037)	0.949 (0.882–1.020)	1.048 (1.016–1.081) **	0.978 (0.910–1.052)
Structural integration				
Organizational participation (ref. no)	1.700 (1.658–1.744) ***	1.654 (1.597–1.713) ***	1.807 (1.763–1.851) ***	1.714 (1.635–1.797) ***
Civil engagement (ref. no)	1.535 (1.497–1.574) ***	1.491 (1.428–1.557) ***	1.605 (1.566–1.644) ***	1.554 (1.491–1.619) ***
Local medical insurance (ref. no)	1.064 (1.033–1.095) ***	1.107 (1.033–1.187) ***	1.049 (1.020–1.079) **	1.110 (1.037–1.189) ***
Sociocultural adaptation				
Having local friends (ref. no)	1.146 (1.114–1.179) ***	1.172 (1.110–1.237) ***	1.150 (1.119–1.182) ***	1.149 (1.090–1.210) ***
Self-identity				
Settlement willingness (ref. no)	1.180 (1.141–1.220) ***	1.104 (1.016–1.200) ***	1.172 (1.135–1.211) ***	1.137 (1.059–1.222) ***
The regional development				
Living in the developed regions (ref. the developing regions)		0.339 (0.216–0.533) ***		0.413 (0.289–0.590) ***
Interaction between social integration and the regional development				
Employment × Developed regions		0.900 (0.769–1.053)		0.859 (0.737–1.001)
Having a local house × Developed regions		1.012 (0.860–1.192)		1.111 (0.946–1.306)
Organizational participation × Developed regions		1.145 (1.061–1.235) **		1.088 (0.984–1.202)
Civil engagement × Developed regions		1.120 (1.020–1.229) *		1.104 (1.014–1.203) *
Local medical insurance × Developed regions		0.952 (0.820–1.105)		0.893 (0.773–1.033)
Having local friends × Developed regions		0.968 (0.857–1.093)		1.040 (0.928–1.165)
Settlement willingness × Developed regions		1.155 (0.944–1.413)		1.084 (0.917–1.282)
−2LL	796,670.6	796,618.9	785,689.9	785,640.2

Notes: Model 1a and model 2a present the results of health education on the prevention of ID. Model 1b and Model 2b present the results of health education on the prevention of NCD. In model 1a and model 1b, we added social integration and control variables. In model 2a and model 2b, we added regional development and the interaction term based on model 1. AOR: Adjusted odds ratio; CI: confidence interval; LL: log likelihood; significance level: *** *p* < 0.001, ** *p* < 0.01, and *p < 0.05.

## Supplementary Materials

The following are available online at https://www.mdpi.com/1660-4601/17/18/6524/s1, Table S1: The empty model of multilevel logistic regressions to the utilization of essential public health services.

## Abbreviations

EPHS	essential public health services
ID	infectious diseases
NCD	noncommunicable chronic disease
CMDS	the China Migrants Dynamic Survey
