# Supplementary material for: The Association between Social Integration and Utilization of Essential Public Health Services among Internal Migrants in China: A Multilevel Logistic Analysis
Source: Int J Environ Res Public Health. 2020 Sep 8;17(18):6524. doi: 10.3390/ijerph17186524 (PMC7559733; doi:10.3390/ijerph17186524)
Supplement: Supplementary file 1 [file ijerph-17-06524-s001.zip › ijerph-887831-supplementary.docx]

**Supplementary material：**

**Table 1S The empty model of multilevel logistic regressions to the utilization of essential public health services (N=154,008)**

|  | **Health records** | **Health education** **on** | |  |
| --- | --- | --- | --- | --- |
|  |  | **Prevention of ID** | **Prevention of NCD** | |
| **Fix effect** |  |  |  | |
| Intercept^a^ | -0.874(0.124)*** | -0.672(0.106)*** | -0.482 (0.089)*** | |
| **Random variance** |  |  |  | |
| Residual^a^ | 0.700 (0.090)*** | 0.597(0.0768)*** | 0.497(0.064)*** | |
| -2LL | 803695.9 | 788621.4 | 776107.9 | |
| ICC | 0.178 | 0.153 | 0.131 | |

Notes: a: coefficient and standard errors are shown; ID: infectious diseases; NCD: noncommunicable chronic disease; LL: log likelihood; ICC: intra-class correlation coefficient; Significance level: *** *p*<0.001.
